# Supplementary material for: Disentangling metabolic functions of bacteria in the honey bee gut
Source: PLoS Biol. 2017 Dec 12;15(12):e2003467. doi: 10.1371/journal.pbio.2003467 (PMC5726620; doi:10.1371/journal.pbio.2003467)
Supplement: S8 Data — (ZIP) [file pbio.2003467.s008.zip › Z-score_plots/85 microbial product Toluene-cis-dihydrodiol.pdf]

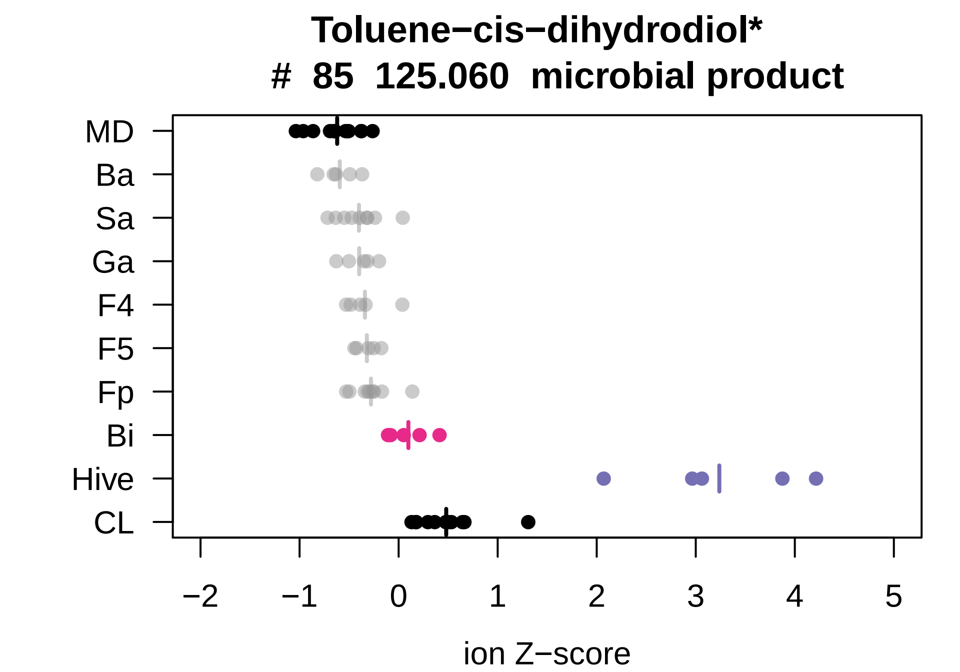
Toluene−cis−dihydrodiol*
85 125.060 microbial product
MD
Ba
Sa
Ga
F4
F5
Fp
Bi
Hive
CL
−2
−1
0
1
2
3
4
5
ion Z−score
